# The Bidirectional Relationship Between Type 2 Diabetes and Metabolic Dysfunction-Associated Steatotic Liver Disease: A Retrospective Cohort Study

**DOI:** 10.7759/cureus.75993

**Published:** 2024-12-19

**Authors:** David M Williams, Jumaina Ali, Jake Cragg, Chin L Ch'ng, Namor W Williams, Jeffrey W Stephens, Thinzar Min

**Affiliations:** 1 Department of Diabetes and Endocrinology, Morriston Hospital, Swansea, GBR; 2 Department of General Medicine, Morriston Hospital, Swansea, GBR; 3 Department of Hepatology, Singleton Hospital, Swansea, GBR; 4 Department of Pathology, Singleton Hospital, Swansea, GBR; 5 Diabetes Research Group, Swansea University Medical School, Swansea, GBR

**Keywords:** a sodium-glucose cotransporter 2 inhibitor, diabetes mellitus type 2, fatty liver disease, glucagon-like peptide-1 receptor agonist, metabolic dysfunction-associated steatotic liver disease, non-alcoholic fatty liver, s: metabolic syndrome

## Abstract

Introduction

Type 2 diabetes (T2D) and metabolic dysfunction-associated steatotic liver disease (MASLD) have shared pathophysiology. We aim to explore associations between these diseases and the impact of T2D therapies on MASLD-related outcomes in a real-world population.

Methods

A retrospective cohort study included 153 patients with biopsy-proven MASLD. Health records were reviewed for biochemical or radiological changes over follow-up and compared by T2D status. The rate of incident T2D was determined, and in those with T2D, the changes over follow-up were compared by prescribed treatment. The statistical significance of changes over follow-up was evaluated by Student's t-test, and logistic regression was undertaken to determine the impact of variables on T2D development.

Results

One hundred and fifty-three patients were included with a mean follow-up of 48.0±22.0 months. Patients with T2D (n=73) were older than patients without T2D (n=80; 56.3 vs 51.9 years, p<0.05). Patients with T2D had a greater stage of hepatic fibrosis (2.6 vs 1.7, p<0.001). Nine (12.3%) patients with T2D and four (5.0%) without T2D died during follow-up (p=0.10). Patients without T2D had greater glycosylated haemoglobin (HbA1c) over follow-up (3.0 mmol/mol, p<0.01), and 21 (26.3%) developed T2D. Patients with T2D treated with sodium-glucose transporter-2 inhibitors (SGLT-2i) and/or glucagon-like peptide-1 receptor analogues (GLP-1RA) had a reduction in FibroScan®-controlled attenuation parameter (-33.7dB/m, p<0.001) but not liver stiffness measure. There were no significant FibroScan® changes in those receiving other treatments.

Conclusions

Patients with T2D had greater hepatic fibrosis, and one in four patients with MASLD developed T2D over four years. Treatment with SGLT-2i and/or GLP-1RA in patients with T2D is associated with improved measures of steatosis but not fibrosis.

## Introduction

The inter-relationship between metabolic dysfunction-associated steatotic liver disease (MASLD) and type 2 diabetes (T2D) is increasingly understood, with major advancements in the recognition of shared aetiologies over the last 20-30 years [1]. Previous meta-analyses observe more than two-fold greater MASLD risk in patients with T2D, with an estimated global prevalence of MASLD, metabolic dysfunction-associated steatohepatitis (MASH), and advanced hepatic fibrosis in patients with T2D of 55.5%, 37.3%, and 4.8%, respectively [2]. Moreover, T2D accelerates MASLD progression to advanced fibrosis [1,2] and is associated with greater all-cause and liver-related mortality [3]. The reverse relationship is also described, with an approximate two-fold greater risk of T2D in patients with MASLD compared to those without MASLD. Moreover, there is an increasing incidence of T2D with progressive fibrosis in patients with MASLD [4]. Previous meta-analyses exploring the associations between T2D and MASLD are often limited by the inclusion of studies with heterogeneous design [4-6].

Developments in pharmacological treatments for MASLD emphasise the association between these disorders [7]. Several drug classes traditionally used to treat T2D are recognised to improve measures of steatohepatitis in MASLD guidelines [8-10], including thiazolidinediones, sodium-glucose co-transporter-2 inhibitors (SGLT-2i) and glucagon-like peptide-1 receptor analogues (GLP-1RAs). These classes are well-established to have cardio-metabolic benefits in patients with T2D, and their use in patients at risk of developing T2D may also reduce the risk of incident T2D. Nonetheless, whilst clinical trial data to date consistently show that pioglitazone [11], SGLT2i [12], and GLP-1RAs [13-15] improve histological or non-invasive measures of steatohepatitis, they do not improve measures of hepatic fibrosis. This is important, as fibrosis is associated with poor outcomes in patients with MASLD [16].

The clinical importance of this relationship is highlighted by the growing prevalence and burden of these disorders. Indeed, in our practice in Wales and internationally, the prevalence of diabetes continues to grow. Moreover, the prevalence of MASLD has grown 10-fold in Wales over the last 20 years, and its relative population burden will soon overtake alcohol-related liver disease [17]. Further investigation into the relationship between the disorders may uncover shared aetiologies or missed treatment opportunities in these patient groups with high cardiometabolic risk.

In this manuscript, we explore the relationship between MASLD and T2D in a real-world population. We aimed to determine any differences in the clinical characteristics, non-invasive test observations, or pathological measures of MASLD between those living with or without T2D and to compare changes in clinical, biochemical, or FibroScan® variables over follow-up. We aimed to describe the changes in glycaemic control and the risk of developing T2D over follow-up in those living without T2D at the time of MASLD diagnosis. Finally, we aimed to evaluate the real-world impact of using SGLT2i and/or GLP-1RA for the treatment of T2D on non-invasive measures of MASLD and patient outcomes over follow-up.

## Materials and methods

Study design

**Figure 1:**
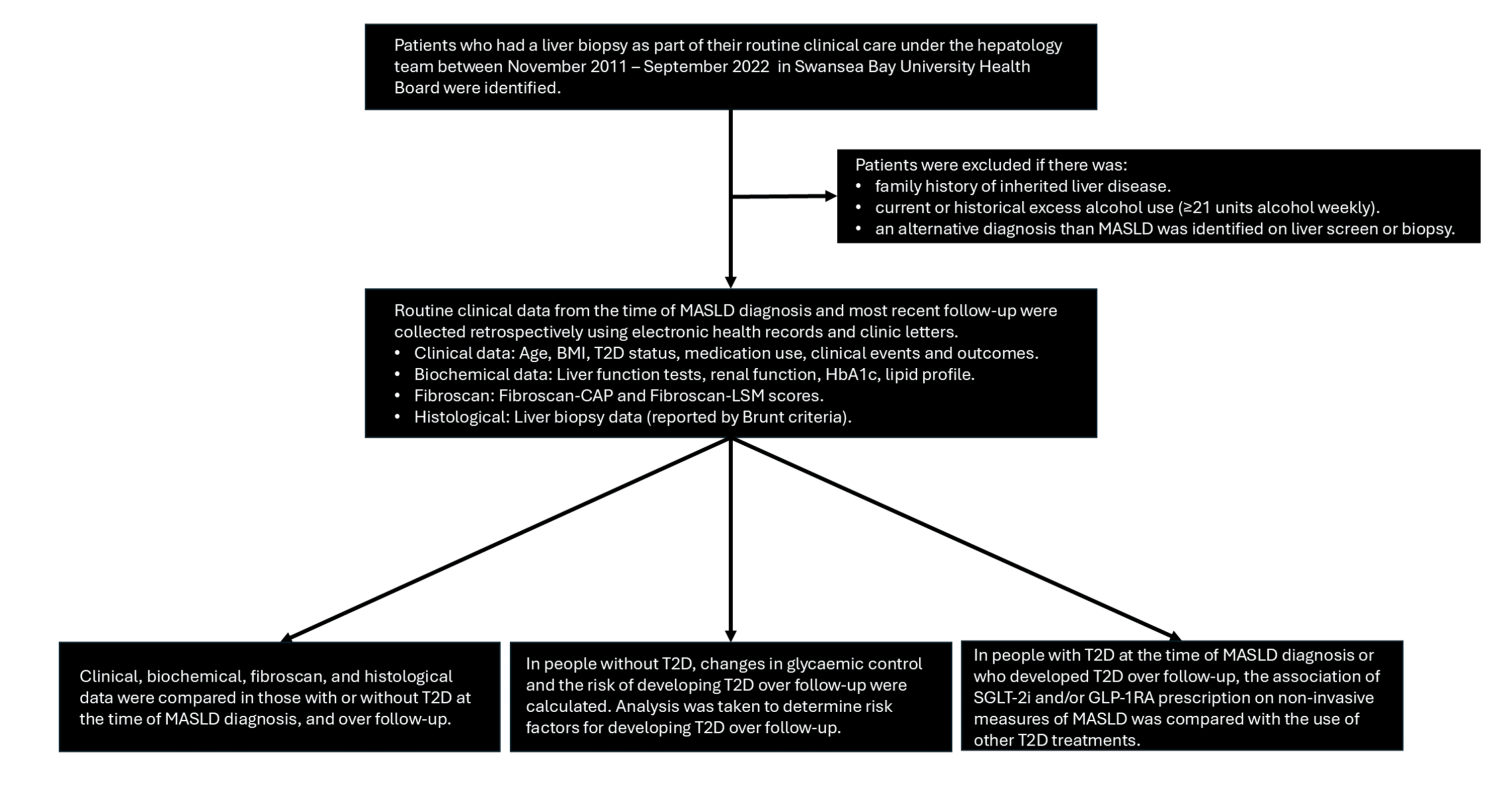
Study design BMI - body mass index; CAP - controlled attenuation parameter; GLP-1RA - glucagon-like peptide-1 receptor analogue; LSM - liver stiffness measure; MASLD - metabolic dysfunction-associated steatotic liver disease; SGLT2i - sodium-glucose co-transporter-2 inhibitor; T2D - type 2 diabetes; HbA1c - glycosylated haemoglobin

This was a retrospective cohort study of patients with biopsy-confirmed MASLD managed in Swansea Bay University Health Board (Morriston Hospital, Neath Port Talbot Hospital, or Singleton Hospital). The study design is presented in Figure 1.

Subjects

Patients included were typically referred to hepatology services with deranged liver enzymes or following observation of incidental fatty or fibrotic liver changes on abdominal imaging. Patients included in the analysis had liver fat identified on imaging and/or liver biopsy, and alternative causes of hepatic steatosis other than MASLD were excluded as part of their clinical care under the hepatology team. All patients had a 'liver screen' to exclude viral hepatitis (hepatitis B and hepatitis C serology), autoimmune hepatitis (anti-nuclear antibodies, anti-smooth muscle antibodies, anti-mitochondrial antibodies), and other metabolic causes (ferritin, and if relevant, alpha-1-antitrypsin or caeruloplasmin levels). Patients did not drink more than 21 units of alcohol weekly at the time of assessment or have a previous history of excess alcohol use, nor a family or personal history of inherited liver disease. All histopathological samples obtained from liver biopsy were reported using the Brunt criteria [18,19]. Patients were deemed to have T2D if their glycosylated haemoglobin (HbA1c) was ≥48 mmol/mol (6.5%) or if it was listed in their medical history. Pre-diabetes was established if HbA1c was 42-47 mmol/mol (6.0 - 6.5%), and a normal HbA1c was defined as ≤41 mmol/mol (5.9%).

Data collection

Electronic health records, including clinic letters, referral letters, and biochemical, radiological, or histopathological tests undertaken for clinical purposes, were reviewed. Routinely collected clinical data, biochemical data, and radiological data were collected from the time of liver biopsy and compared to the most recent follow-up. Radiological data included the controlled attenuation parameter (CAP) and liver stiffness measure (LSM) scores which were determined by FibroScan®. Missing histopathological data were reviewed by a consultant histopathologist working in Swansea. The Welsh Index of Multiple Deprivation (WIMD) was used to measure subjects' deprivation based on postcode data. Those with a lower WIMD have greater deprivation, and those with a higher WIMD have relatively less deprivation.

Funding and ethical approval

No additional funding was received to undertake the study. The study was part of a local service evaluation conducted in collaboration between the hepatology team in Swansea and the diabetes research group at Swansea University Medical School. Data were collected retrospectively as part of routine clinical practice and presented anonymously; therefore, ethical approval was not required.

Statistical analysis

Statistical analysis was performed using SPSS, version 29 (IBM Inc., Armonk, US). Categorical data are presented as the number (%), and statistical significance was determined using a Chi-squared test. Shapiro-Wilk test was used to examine the normality of data distribution. Continuous data are presented as the mean ± standard deviation, and statistical significance was evaluated by Student's t-test. The association between risk factors (age, BMI, deprivation index, baseline HbA1c) and development of T2D in patients with MASLD were examined using logistic regression analysis. Statistical significance was determined using paired data and usually at p<0.05. Patients with missing data were excluded from the analysis, and the number of participants with available paired data is indicated in the tables presented.

## Results

Patient characteristics

A total of 153 patients with MASLD were included. Table 1 presents the clinical characteristics of the cohort and compares baseline data between patients with or without T2D.

**Table 1:** Patient characteristics at the time of liver biopsy Cohort characteristics, comparing the characteristics of patients with or without T2D at the time of liver biopsy. Categorical data are presented as the number (%), and continuous data are presented as the mean±standard deviation. Statistical significance of differences at baseline between patients with or without T2D is presented by *p<0.05, **p<0.01, ***p<0.001. ALP - alkaline phosphatase; ALT - alanine transaminase; APRI - aspartate transaminase to platelet ratio index; AST - aspartate transaminase; BARD - BMI-AST/ALT ratio-diabetes mellitus; BMI - body mass index; CAP - controlled attenuation parameter; eGFR - estimated glomerular filtration rate; FIB-4 - fibrosis-4; GGT - gamma-glutamyl transferase; HbA1c - glycated haemoglobin; HDL - high-density lipoprotein; HSI - hepatic steatosis index; LDL - low-density lipoprotein; LSM - liver stiffness measure; NAS - non-alcoholic fatty liver disease activity score; NFS - non-alcoholic fatty liver disease fibrosis score; NR - normal range; T2D - type 2 diabetes; TC - total cholesterol; TG - triglyceride; WIMD - Welsh Index of Multiple Deprivation

Characteristic	Total cohort (n=153)	Patients with T2D (n=73)	Patients without T2D (n=80)
Age (years)	54.0±11.5	56.3±10.2	51.9±12.3^*^
Male sex	80 (52.3%)	41 (56.2%)	39 (48.8%)
WIMD decile	4.7±2.9	4.6±2.7	4.9±3.1
BMI (kg/m^2^)	36.9±7.0	37.8±6.8	36.0±7.2
NAS	3.9±1.5	3.9±1.5	3.9±1.5
Steatosis	2.0±0.8	2.0±0.8	1.9±0.9
Inflammation	1.0±0.6	1.1±0.6	1.0±0.6
Ballooning	0.9±0.7	0.9±0.7	1.0±0.7
Fibrosis	2.1±1.5	2.6±1.4	1.7±1.4^***^
F0	30 (19.6%)	7 (9.6%)	23 (28.8%)^**^
F1	33 (21.6%)	13 (17.8%)	20 (25.0)
F2	13 (8.5%)	6 (8.2%)	7 (8.7%)
F3	43 (28.1%)	21 (28.8%)	22 (27.5%)
F4	34 (22.2%)	26 (35.6%)	8 (10.0%)^***^
FibroScan® – CAP (dB/m)	346.4±52.5	358.3±35.0	335.6±62.7^*^
FibroScan® – LSM (kPa)	16.2±11.2	17.7±11.5	14.8±10.9
<8.2kPa (F0-1)	28 (18.3%)	12 (16.4%)	16 (20.0%)
8.2-9.7kPa (F2)	17 (11.1%)	7 (9.6%)	10 (12.5%)
9.7-13.6kPa (F3)	29 (19.0%)	9 (12.3%)	20 (25.0%)^*^
>13.6kPa (F4)	62 (40.5%)	37 (50.7%)	25 (31.3%)^*^
Missing FibroScan® - LSM data	17 (11.1%)	8 (11.0%)	9 (11.2%)
ALT (U/L) [NR <41]	58.1±43.4	55.3±38.1	60.7±48.0
ALP (U/L) [NR 30-130]	95.1±44.5	96.3±43.6	94.0±45.6
AST (U/L) [NR <40]	49.1±53.9	41.6±24.0	54.3±67.2
GGT (U/L) [NR <60]	152.2±211.4	112.1±82.2	188.2±277.5
Platelets (x10^9^/L) [NR 150-400]	226.5±72.2	214.6±72.2	237.4±71.0^*^
TC (mmol/L)	4.8±1.3	4.6±1.3	5.1±1.4^*^
LDL (mmol/L)	2.8±1.2	2.4±0.9	3.1±1.3^***^
HDL (mmol/L) [NR >1.0]	1.2±0.3	1.1±0.3	1.2±0.4^*^
TG (mmol/L) [NR <2.0]	2.4±1.8	2.7±2.1	2.2±1.3
Statin prescription	60 (39.2%)	39 (53.4%)	21 (26.3%) ^***^
HbA1c (mmol/mol) [NR <48]	51.6±18.6	64.4±17.3	37.6±4.9^***^
Creatinine (µmol/L) [NR 58-110]	76.8±19.0	78.5±21.8	75.1±16.0
eGFR (mL/min/1.73m^2^) [NR >90]	85.3±21.6	84.1±21.9	86.5±21.3
HSI	49.2±8.1	50.3±7.8	48.4±8.3
AST:ALT ratio	1.0±0.7	1.0±0.6	1.0±0.7
BARD score	2.3±1.2	3.0±1.1	1.9±1.1^***^
FIB-4 score	2.0±2.1	2.0±1.5	2.0±2.4
NFS	-0.9±2.1	0.2±1.5	-1.7±2.2^***^
APRI	0.8±1.0	0.7±0.6	0.9±1.2

Comparing outcomes in patients with or without T2D during follow-up

Patients were followed up for a mean of 48.0±22.1 months since the liver biopsy, and this did not differ significantly between patients with T2D or without T2D (45.0±20.6 vs 50.8±23.3 months, p=0.12). Changes over follow-up are presented in Table 2 and compared by T2D status. The difference in the change in FibroScan®-LSM score between patients with T2D compared to patients without T2D was not significant (-3.6 vs +0.9kPa, p=0.11).

**Table 2:** Differences in clinical characteristics observed during follow-up in patients with or without T2D Comparing differences in clinical characteristics over follow-up in patients with or without T2D. Continuous data are presented as the mean±standard deviation. The statistical significance of differences in these clinical variables in patients with or without T2D at follow-up from baseline are presented by *p<0.05, **p<0.01, ***p<0.001. ALP - alkaline phosphatase;  ALT - alanine transaminase; APRI - aspartate transaminase to platelet ratio index; AST - aspartate transaminase; BARD - BMI-AST/ALT ratio-diabetes mellitus; BMI - body mass index; CAP - controlled attenuation parameter; eGFR - estimated glomerular filtration rate; FIB-4 - fibrosis-4; GGT - gamma-glutamyl transferase; HbA1c - glycated haemoglobin; HDL - high-density lipoprotein; HSI - hepatic steatosis index; LDL - low-density lipoprotein; LSM - liver stiffness measure; NAS - non-alcoholic fatty liver disease activity score; NFS - non-alcoholic fatty liver disease fibrosis score; T2D - type 2 diabetes; TC - total cholesterol; TG - triglyceride

Characteristic	Patients with T2D (n=73)			Paired data	Patients without T2D (n=80)		Paired data
	Baseline		Follow-up		Baseline	Follow-up	
BMI (kg/m^2^)	37.4±6.6	35.8±6.7^***^		n=62	35.8±7.2	34.7±6.2^*^	n=52
FibroScan® – CAP (dB/m)	363.7±25.1	333.7±43.4^***^		n=38	334.5±57.7	313.0±50.3^*^	n=42
FibroScan® – LSM (kPa)	17.2±13.1	13.6±6.9		n=38	13.0±6.9	13.9±13.5	n=44
ALT (U/L) [NR <41]	55.1±38.3	37.1±22.6^***^		n=68	61.3±50.4	46.5±39.2^*^	n=70
ALP (U/L) [NR 30-130]	95.8±44.0	106.3±39.2^*^		n=68	94.3±45.8	105.4±79.3^*^	n=70
AST (U/L) [NR <40]	44.2±23.0	33.5±15.0^*^		n=17	61.9±99.0	40.3±20.5	n=27
GGT (U/L) [NR <60]	144.8±54.2	134.3±106.5		n=4	108.3±143.0	71.5±30.8	n=4
Platelets (x10^9^/L) [NR 150-400]	216.1±67.7	221.1±93.0		n=66	237.4±70.8	220.8±73.6^**^	n=70
TC (mmol/L)	4.6±1.3	4.2±1.0^*^		n=65	5.2±1.5	4.8±1.5^*^	n=51
LDL (mmol/L)	2.4±0.8	2.2±1.0^*^		n=65	3.2±1.3	2.7±1.3^**^	n=51
HDL (mmol/L) [NR >1.0]	1.1±0.3	1.1±0.3		n=65	1.2±0.4	1.4±0.5	n=51
TG (mmol/L) [NR <2.0]	2.8±2.2	2.5±1.4		n=65	2.2±1.3	2.1±1.1	n=51
HbA1c (mmol/mol) [NR <48]	64.6±17.0	62.5±19.1		n=67	37.6±4.9	40.6±8.5^**^	n=53
Creatinine (µmol/L) [NR 58-110]	78.4±22.0	91.1±58.8^*^		n=68	74.4±15.6	75.2±14.2	n=71
eGFR (mL/min/1.73m^2^) [NR >90]	84.0±21.5	79.7±30.2		n=68	86.9±21.5	83.4±17.9	n=71
HSI	51.7±7.8	52.5±15.1		n=16	48.2±11.3	43.3±8.8	n=8
AST:ALT ratio	1.0±0.4	1.2±1.0		n=17	0.9±0.7	1.0±0.7	n=27
BARD score	3.2±1.0	3.4±0.9		n=18	1.5±1.0	1.9±1.1	n=28
FIB-4 score	1.9±0.8	2.6±2.6		n=16	1.7±2.3	2.1±3.2	n=26
NFS	0.5±1.4	0.8±2.2		n=17	-2.5±2.3	-1.8±2.3^*^	n=27
APRI	0.7±0.5	0.6±0.4		n=16	1.0±1.7	0.7±0.8	n=26

Table 3 presents the differences in clinical outcomes over follow-up, comparing rates of decompensated liver disease, hepatocellular carcinoma, and all-cause mortality. All four deaths in patients without T2D were related to liver disease, whilst patients with T2D died from various causes (liver disease (n=1), cardiovascular (CV)disease (n=3), malignancy (n=3), and sepsis (n=2).

**Table 3:** Clinical outcomes comparing patients with or without T2D Comparing rates of decompensated liver disease, hepatocellular carcinoma, and all-cause mortality by T2D status over follow-up. Differences between the groups did not reach statistical significance. Categorical data are presented as the number (%). HCC - hepatocellular carcinoma; T2D - type 2 diabetes

Clinical outcome	Overall cohort (n=153)	Patients with T2D (n=73)	Patients without T2D (n=80)
Jaundice	16 (10.5%)	5 (6.9%)	11 (13.8%)
Hepatic encephalopathy	6 (3.9%)	1 (1.4%)	5 (6.3%)
Ascites	9 (5.9%)	2 (2.7%)	7 (8.8%)
Coagulopathy	8 (5.2%)	2 (2.7%)	6 (7.5%)
HCC	0 (0.0%)	0 (0.0%)	0 (0.0%)
Death	13 (8.5%)	9 (12.3%)	4 (5.0%)

Over follow-up, eight (11.0%) patients with pre-existing T2D and 14 (17.5%) without T2D were initiated on a statin (p=0.25). At the most recent visit, 47 (64.4%) patients with pre-existing T2D versus 35 (43.8%) without T2D were prescribed a statin (p=0.01). 

The risk of developing prediabetes or T2D in patients with MASLD

**Figure 2:**
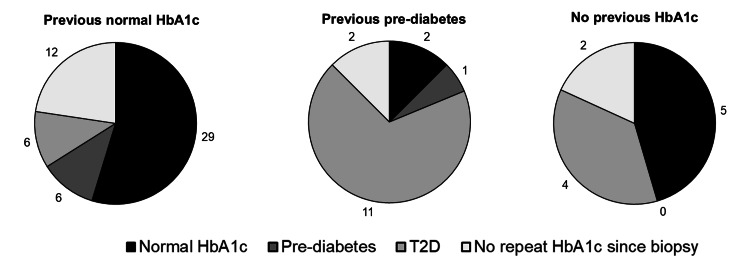
Changes in diabetes status over follow-up based on the patients diabetes status at the time of MASLD diagnosis T2D - type 2 diabetes; HbA1c - glycated haemoglobin; MASLD - metabolic dysfunction-associated steatotic liver disease

At liver biopsy, 80 (52.3%) patients did not have T2D. Of these, 53 (66.3%) had a normal HbA1c, 16 (20.0%) had prediabetes, and 11 (13.7%) had no previous HbA1c. By the most recent clinic visit, 21 (26.3%) patients were diagnosed with T2D, seven (8.7%) had prediabetes, and 52 (65.0%) had a normal HbA1c or no-repeat HbA1c. Of those who developed T2D, 11 (52.4%) had prediabetes, six (28.6%) had a previously normal HbA1c, and four (19.0%) had no previous HbA1c. The changes in the frequency of prediabetes or T2D diagnosis at follow-up presented by the patient's baseline diabetes status can be seen in Figure 2. 

**Figure 3:**
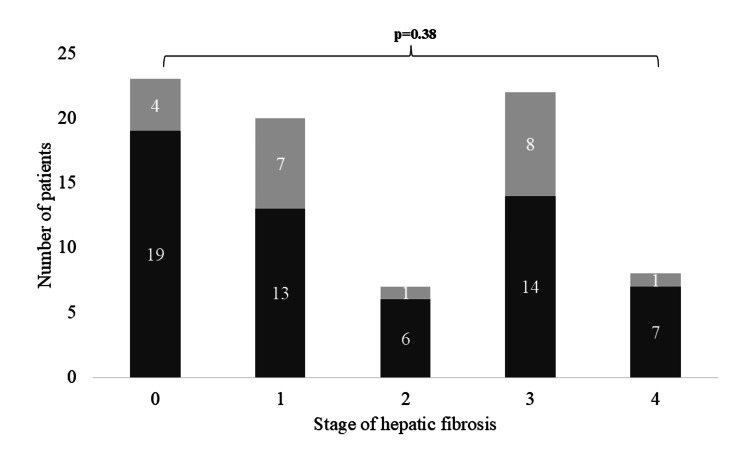
The risk of developing T2D over follow-up by stage of hepatic fibrosis The stacked bar chart presents the risk of developing T2D over follow-up by stage of hepatic fibrosis in patients without pre-existing T2D. The light grey illustrates those who did develop T2D, and dark grey indicates those who did not. T2D - type 2 diabetes

The only difference in the characteristics of patients who developed T2D was greater HbA1c at biopsy (42.2±3.8 vs 36.1±4.2 mmol/mol, p<0.001). Baseline HbA1c was associated with the risk of developing T2D (OR: 2.1, 95% CI: 1.3-3.4). BMI, age, and deprivation index were not associated with the risk of developing T2D. There was no significant difference in the stage of hepatic fibrosis or NAS between those who did or did not develop T2D. Similarly, there was no association between the stage of fibrosis and the risk of developing T2D, as presented in Figure 3.

The impact of SGLT-2i and/or GLP-1RAs on MASLD in patients with T2D

**Figure 4:**
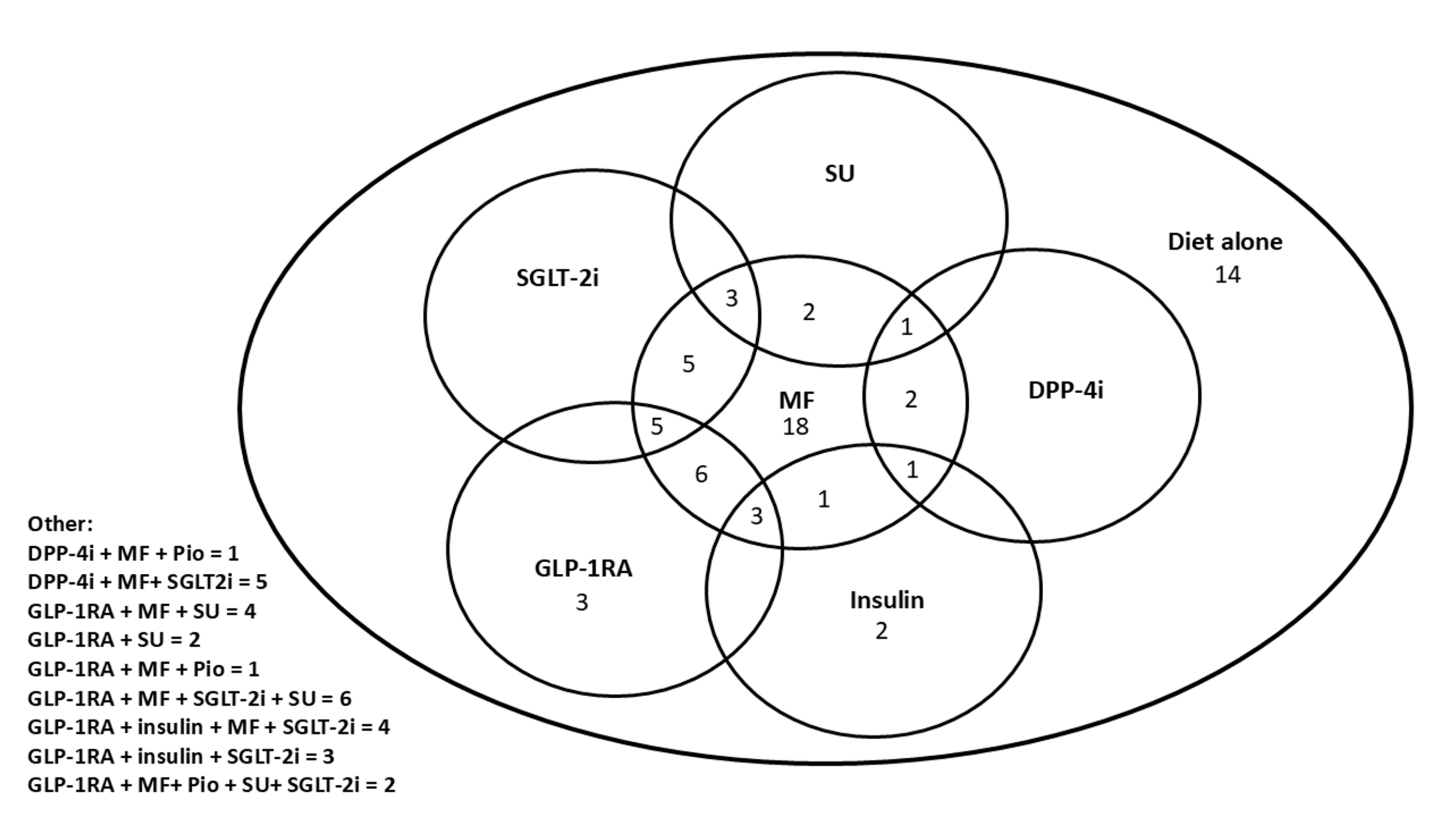
Treatments used for T2D in patients with MASLD at the most recent clinic visit DPP-4i - dipeptidyl peptidase-4 inhibitor; GLP-1RA - glucagon-like peptide-1 receptor agonists; MF - metformin; Pio - pioglitazone; SGLT-2i - sodium-glucose co-transporter-2 inhibitor; SU - sulphonylurea

Ninety-four patients were included (73 with T2D at biopsy, 21 who developed T2D over follow-up), with a mean age of 56.5±10.5 years, BMI 37.5 kg/m^2^, and HbA1c 63.6±21.6 mmol/mol. At biopsy, these patients had a NAS of 4.0±1.5 and a fibrosis stage of 2.4±1.4. The medications prescribed to this group for T2D are presented in Figure 4.

Patients were followed up on treatment over a mean of 4.5±1.8 years. Changes in the observed measures compared by the prescribed T2D treatment in patients with T2D are shown in Table 4.

**Table 4:** Changes over follow-up by prescribed T2D treatment Changes in characteristics over follow-up in patients with T2D prescribed different T2D treatments. Continuous data are presented as the mean±standard deviation. The significance of changes over follow-up is presented by *p<0.05, **p<0.01, ***p<0.001. ALP - alkaline phosphatase; ALT - alanine transaminase; APRI - aspartate transaminase-to-platelet ratio index; AST - aspartate transaminase; BMI - body mass index; CAP - controlled attenuation parameter; eGFR - estimated glomerular filtration rate; FIB-4 - fibrosis-4; GLP-1RA - glucagon-like peptide-1 receptor analogue; HbA1c - glycated haemoglobin; HDL - high-density lipoprotein; LDL - low-density lipoprotein; LSM - liver stiffness measure; NFS - non-alcoholic fatty liver disease fibrosis score; SGLT-2i - sodium-glucose co-transporter-2 inhibitor; T2D - type 2 diabetes; TC - total cholesterol; TG - triglyceride

Variable	SGLT2i±GLP-1RA (baseline, n=52)	SGLT2i±GLP-1RA (follow-up, n=52)	Paired data	Other (baseline, n=42)	Other (follow-up, n=42)	Paired data
Weight (kg)	113.5±24.8	108.0±26.0^***^	n=50	103.0±23.9	99.0±23.2^*^	n=38
BMI (kg/m^2^)	38.5±7.3	37.1±8.0	n=50	36.1±7.2	32.7±9.7^*^	n=38
TE-CAP (dB/m)	365.2±26.5	331.5±33.0^***^	n=17	359.5±32.5	343.6±45.9	n=28
TE-LSM (kPa)	15.0±6.8	13.3±7.5	n=17	15.8±14.4	13.8±9.0	n=29
ALT (U/L) [NR <41]	63.4±45.2	40.0±28.6^***^	n=48	59.6±52.2	39.0±26.8^**^	n=40
ALP (U/L) [NR 30-130]	99.8±41.4	107.7±42.0	n=48	95.5±53.9	115.6±98.7^*^	n=40
AST (U/L) [NR <40]	41.0±27.2	28.3±13.5	n=11	87.2±160.2	33.4±16.2	n=10
GGT (U/L) [NR <60]	223.1±66.8	230.4±99.7	n=46	234.4±56.9	68.1±10.8	n=40
Platelets (x10^9^/L) [NR 150-400]	4.4±0.9	3.9±0.9^**^	n=48	4.8±1.4	4.8±1.6	n=37
TC (mmol/L)	2.4±0.9	1.9±0.8^***^	n=40	2.8±1.2	2.5±1.1	n=35
LDL (mmol/L)	1.1±0.3	1.1±0.3	n=48	1.2±0.4	1.3±0.4	n=37
HDL (mmol/L) [NR >1.0]	2.7±1.9	2.4±1.5	n=48	2.1±1.1	2.4±1.7	n=37
TG (mmol/L) [NR <2.0]	71.6±22.7	60.5±14.5^**^	n=48	53.1±14.8	54.6±16.5	n=37
HbA1c (mmol/mol) [NR <48]	75.3±21.0	90.3±64.5^*^	n=50	75.6±17.5	82.0±30.0	n=40
Creatinine (µmol/L) [NR 58-110]	85.9±20.6	80.4±26.0	n=48	86.1±20.5	81.9±28.9	n=40
FIB-4 score	1.5±1.0	1.9±2.1	n=11	2.0±2.3	1.8±2.1	n=10
NFS	0.4±1.3	0.6±1.3	n=11	-0.3±2.2	0.0±2.6	n=10
APRI	0.6±0.5	0.5±0.4	n=11	1.3±2.5	0.5±0.3	n=10

At follow-up, 39 (75.0%) patients prescribed SGLT-2i and/or GLP-1RA were prescribed a statin versus 21 (50.0%) prescribed other treatments (p<0.05). In those prescribed SGLT-2i and/or GLP-1RA, four out of 52 (7.7%) died, versus five out of 42 (11.9%) in those prescribed other treatments (p=0.48).

## Discussion

In this study, we aimed to explore the bidirectional relationship between MASLD and T2D in a cohort of patients with biopsy-confirmed MASLD. There was a high frequency of T2D in the cohort at the time of liver biopsy, affecting almost half of the group. This is around six times higher than the prevalence of T2D in Wales [20]. However, some studies previously reported that the addition of further risk factors to MASLD can increase the risk of T2D up to 14-fold, such as obesity and insulin resistance [21]. This cohort had a mean BMI of 36.9 kg/m^2^, inferring a greater risk of T2D. However, it is difficult to establish a causative association between these disorders in this cross-sectional observation. Nonetheless, our findings do highlight the complicated relationship between these disease states given the high frequency of T2D in this cohort, the greater stage of MASLD-related fibrosis observed in those living with T2D, the high risk of developing T2D over the observed four-year follow-up and the improvements in some non-invasive measures of MASLD with the use of SGLT2i and/or GLP-1RA.

We observed that patients with T2D had a significantly greater mean fibrosis score at liver biopsy and a greater proportion of patients with stage F4 liver fibrosis on liver biopsy or equivalent to F4 liver fibrosis using the FibroScan®-LSM score compared to those without T2D, despite similar measures of hepatic steatosis, inflammation, and ballooning. There are several possible reasons for this. Firstly, T2D is a major risk factor for accelerated hepatic fibrosis [1,2], which may be independent of other histological measures. Here, T2D-specific mechanisms such as dysfunctional adipose tissue, genetic factors, and the impact of glucotoxicity may play a role [22]. Secondly, the greater age of patients with T2D in this cohort may reflect that these patients had a greater duration of unrecognised disease, causing a greater duration of hepatic insult and, therefore, progressive fibrosis. Therefore, this result may be somewhat predictable, and their greater age may be a confounder in this analysis. Certainly, there are observations of a growing burden of MASLD and T2D in younger patients, including children [23]. Moreover, given the frequency of hepatic steatosis on abdominal imaging in patients with T2D, there may be a mistaken perception that MASLD is a benign disease in this cohort, resulting in clinical inaction. Thirdly, factors unaccounted for in this study may be important, such as medical co-morbidities (e.g., hypothyroidism, hypertension) or smoking status, data for which are limited in this retrospective study.

At the time of biopsy, patients with T2D had a greater FibroScan®-CAP score despite similar hepatic steatosis scores on histology. This may reflect a limitation of FibroScan® in patients with higher BMI, especially in those with a BMI greater than 45 kg/m^2^ [24]. Indeed, the mean BMI in our cohort was 36.9 kg/m^2^. In this cohort, 20 (13.1%) had a BMI greater than 45 kg/m^2^, with a similar proportion in those with or without T2D (15.1% vs 11.3%, p=0.48). Similarly, this limitation may explain the lack of significance towards a greater FibroScan®-LSM observed in patients with T2D despite a significantly greater liver fibrosis score at biopsy.

Patients with T2D had a greater rate of statin prescription than those without T2D, which would explain the better lipid profiles in those with T2D. Greater statin prescription in those with T2D was expected, given that this group is, by their definition, more likely to have greater CV risk [25]. Importantly, the greater rate of statin prescription is highly unlikely to explain the greater degree of hepatic fibrosis in this group, given previous studies have not demonstrated associations between statin use and hepatic fibrosis risk [26] and their use for dyslipidaemia and CV risk management in patients with MASLD is supported by current international guidance [8-10].

Despite the greater use of statins and improved lipid profiles, patients with T2D were more than twice as likely to die over follow-up, which may reach statistical significance with a greater duration of follow-up and number of events. All four patients who died without T2D succumbed to liver disease, whilst patients who died with T2D had other causes of death, including CV disease or malignancy. Indeed, others have previously observed that patients with T2D have greater mortality, which is largely attributable to excess CV and malignant disease [1,2,27,28]. However, the trend to reduced decompensated liver disease and liver-related mortality was unexpected, as this cohort had a greater degree of hepatic fibrosis, inferring more advanced liver disease and decompensation risk, and T2D is usually considered a risk factor for MASLD decompensation. Nonetheless, given the small number of events and lack of statistical significance in this cohort, conclusions are limited.

There was a significant increase in the mean HbA1c in patients without T2D over four years, and one in four developed T2D over observed follow-up. In those with a normal HbA1c prior to MASLD diagnosis, six out of 53 (11.3%) developed prediabetes, and six out of 53 (11.3%) developed T2D. Of those with prediabetes at the time of MASLD diagnosis, 11 out of 16 (68.8%) developed T2D. Moreover, only baseline HbA1c was found to be associated with the development of T2D in the cohort. One previous meta-analysis found that patients with MASLD have a 2.2-fold greater risk of developing T2D than patients without MASLD [4]. In this meta-analysis, 5.6% of over half a million patients with (30.8%) or without (69.2%) MASLD developed T2D over five years. The finding of a mean annual incidence of T2D of around 6.5% in our cohort is greater than the previously presented risk, though comparisons are limited by variable definitions of MASLD in the studies included in the meta-analysis and limited comparisons of other risk factors for T2D, such as BMI.

Given the close relationship between T2D and MASLD, we were disappointed that 13.7% of patients did not have an HbA1c test before liver biopsy, as this is, of course, a potential treatment target that can reduce MASLD progression. A finding of T2D in the context of MASLD supports the use of a greater breadth of therapies, such as SGLT-2i and/or GLP-1RAs [8]. However, we observed infrequent use of vitamin E or pioglitazone in the overall cohort, and there was inconsistent use of SGLT-2i or GLP-1RAs in patients with T2D. In those in whom these drugs were used, there were significant improvements in FibroScan®-CAP score, bodyweight, alanine transaminase (ALT), lipid profiles, and HbA1c. However, other T2D treatments improved bodyweight and ALT only, and to a lesser extent than seen with SGLT-2i and/or GLP-1RAs. Several studies report improved non-invasive measures of MASLD associated with SGLT-2i [12]. Furthermore, GLP-1RA use improves invasive and non-invasive measures of steatohepatitis in clinical trials [13,14]. In our study, similar findings are observed by improved FibroScan®-CAP and reduced ALT. Like in clinical trials [12-14], we did not see improved measures of liver fibrosis over follow-up. Unfortunately, we were not able to undertake further sub-analysis comparing those who were prescribed SGLT2i alone, GLP-1RA alone, or both SGLT2i and GLP-1RA with those prescribed other T2D treatments because the number of patients in each of these subgroups with paired data were limited. This may have been of interest given the particularly significant impact of GLP-1RAs on MASLD [13,14].

The impact of these drugs on bodyweight, HbA1c, and other metabolic measures is well-established in patients with T2D [29,30]. The significant reduction in HbA1c was only observed in those prescribed SGLT-2i and/or GLP-1RA, but this is likely a consequence of their higher baseline HbA1c and may be confounded by the variable therapies prescribed to the 'other treatment' group. Likewise, the apparent impact of these medicines on lipids may be multifactorial, given those prescribed SGLT-2i and/or GLP-1RA also had greater statin use.

Ultimately, supporting primary and secondary care colleagues in considering the use of these therapies when appropriate may support the concurrent treatment of T2D and MASLD and address their excess CV risk. The use of these medicines may reduce the incidence of T2D in such cohorts. Likewise, screening for MASLD in patients with T2D may identify those at risk of advanced fibrosis and permit meaningful therapy and liver disease monitoring [8-10]. Nonetheless, clinical inertia in the management of T2D and MASLD in practice can restrain therapeutic intervention. A multidisciplinary team of specialist doctors, nurses, dieticians, physiotherapists, and psychologists may better support the management of this frequently under-treated patient group.

Study limitations

There are several limitations to this study. Firstly, the retrospective design relied upon already collected and documented routine clinical data, which may be prone to the biases typically afflicting retrospective studies. Greater duration of follow-up or inclusion of more patients may uncover further differences in clinical outcomes and characteristics between those with or without T2D. There was limited information on secondary care records to confirm the duration of medication use and specific dietetic support employed. There was a lack of a control group without MASLD, which may offer further comparative insights. Subgroup analyses comparing those taking specific drug classes (e.g., SGLT-2i versus GLP-1RA) were limited by patient numbers.

## Conclusions

In this study, patients with T2D and MASLD were significantly older and had greater liver fibrosis on histopathological assessment, regardless of similar measures of hepatic steatosis, inflammation, and ballooning. Over follow-up, 21 (26.3%) patients without T2D developed T2D, of whom most had pre-existing prediabetes. The use of recommended pharmacotherapies for MASLD in the group was low, though, in those treated with SGLT2i and/or GLP-1RA, there was some evidence to support improved hepatic steatosis. Greater clinician awareness of MASLD in patients with T2D may support more targeted therapy use.
